# Comparative Transcriptome and Proteome Analysis of Heat Acclimation in Predatory Mite *Neoseiulus barkeri*

**DOI:** 10.3389/fphys.2020.00426

**Published:** 2020-04-29

**Authors:** Chuan Bei Tian, Ya Ying Li, Ji Huang, Wen Qiang Chu, Zi Ying Wang, Huai Liu

**Affiliations:** Key Laboratory of Entomology and Pest Control Engineering, College of Plant Protection, Southwest University, Chongqing, China

**Keywords:** *Neoseiulus barkeri*, heat acclimation, transcriptome, proteome, heat shock protein, metabolism

## Abstract

In our previous study, we reported a high temperature adapted strain (HTAS) of the predatory mite *Neoseiulus barkeri* was artificially selected via a long-term heat acclimation (35°C) and frequent heat hardenings. To understand the molecular basis of heat acclimation, ‘omics’ analyses were performed to compare the differences between HTAS female adults to conventional strain (CS) at transcriptional and translational levels. We obtained a total of 5,374 differentially expressed genes and 500 differentially expressed proteins. Among them, 119 transcripts had concurrent transcription and translation profiles. It’s conserved that some processes, such as high expression of heat shock protein (HSP) genes, involved in heat tolerance of transcriptome analyses, while many protective enzymes including glutathione S-transferase, superoxide dismutase, peroxidase, and cytochrome P450 displayed down-regulated expression. KEGG analysis mapped 4,979 and 348 differentially expressed genes and proteins, to 299 and 253 pathways, respectively. The mitogen-activated protein kinases (MAPK) signaling pathway may provide new insights for the investigation of the molecular mechanisms of heat tolerance. Correlation enriched pathways indicated that there were four pathways associated with heat acclimation involving in energy metabolism and immunity. In addition, the expression patterns of ten randomly selected genes including HSP were consistent with the transcriptome results obtained through quantitative real-time PCR. Comparisons between transcriptome and proteome results indicated the upregulation of HSPs and genes participated in ATP production, immunity and energy metabolism process. A majority of antioxidant-related genes and detoxication-related genes were down-regulated suggesting a fitness cost of heat acclimation. Our results demonstrated that heat tolerance during a long-time acclimation of *N. barkeri* is a fairly complicated process of physiological regulations. These findings also contribute to a better understanding of the mechanisms of thermal responses of phytoseiid mites which could provide useful information for biological control through natural enemies.

## Introduction

Phytoseiid mites (Acari: Phytoseiidae), as the effective natural enemies of spider mites and small insect pests, are widely used for biological control on fruits, vegetables, and other crops worldwide (Nomikou et al., 2001; Arthurs et al., 2009; McMurtry et al., 2013; Kamel et al., 2018). Both phytoseiid mites and pest mites are tiny organisms, and they are prone to environmental challenges in the field, such as thermal stress (Veerman, 1992; Gotoh et al., 2004), pesticides (Bin Ibrahim and Yee, 2000; Li et al., 2006; Sato et al., 2011), ultraviolet-B radiation (Tachi and Osakabe, 2012; Ghazy et al., 2016) and drought stress (Ferrero et al., 2010). Most studies have proved that phytoseiid mites were more vulnerable than the spider mites under environmental stressors including high temperature (Stavrinides and Mills, 2011) and ultraviolet radiation (Tachi and Osakabe, 2012), while pest mites could endure and survive from them, which caused herbivore natural control disruptions and failures in biological control strategies (Guzman et al., 2016). Climate change may become a crucial limiting factor for biological control in agriculture, since the chances of pests to escape from predator control may increase because vulnerability to rising temperature usually increases with trophic level. Vulnerability to rising temperatures usually increases with trophic level which may cause increasing chances of pest to escape predation in climate change situation (Voigt et al., 2003).

*Neoseiulus barkeri* Hughes (Acari: Phytoseiidae) is an effective natural enemy and important biological control agent for spider mites and several small insect pests (Bonde, 1989; Wu et al., 2014) which has been widely distributed and applied in China (Niu et al., 2014). However, due to their poor adaptability in the field condition, its application in agro-ecosystems is often unsatisfactory. To improve this worrying situation, a high temperature adapted strain (HTAS) of *N. barkeri* was selected from a conventional strain (CS) that maintained at 25 ± 1°C via a long-term heat acclimation and frequent heat hardenings in 2012 (Zhang G. H. et al., 2018). It has been proved that the heat acclimation greatly improved their survival probabilities under heat stress, e.g., the survival probabilities of adult females up to 90% when exposed at 45°C for 2–6 h, indicating a strong tolerance of HTAS *N. barkeri* to heat stress was obtained or developed after heat acclimation. In addition, long-time heat acclimation has also resulted in accelerated growth and developmental rate and reduced total fecundity and longevity which indicated the potential costs on fitness of heat acclimation (Zhang G. H. et al., 2018).

Acclimatory responses (short or long-time) are often considered a multistep process, including detecting environmental cues, transducing signals into a cellular response, and activating certain genes, proteins and metabolites that regulate physiological function to enhance thermotolerance. The physiological adaptation to thermal acclimation has been extensively studied in *Drosophila* and other insects (Kregel, 2002). Acclimation of *Drosophila melanogaster* could induce adaptive changes in metabolic rates and membrane lipids composition, indicating acclimation-related physiological adjustments (Berrigan, 1997; Overgaard et al., 2008). Analysis of proteomic responses induced by heat stress in insects showed many thermal tolerance related proteins involved in iron ion and cell redox homeostasis, carbohydrate and energy metabolism (Colinet et al., 2013), structural elements of the cytoskeleton (Nguyen et al., 2009) and stress-induced signal transduction (Li et al., 2012). However, in spite of the findings and extensive knowledge of thermotolerance mechanism in genetics among many insects, the understanding of acclimation-related physiological adjustments of phytoseiid mites remains poorly studied.

For a more detailed understanding of the thermal acclimatory responses of phytoseiid mites and the physiological difference between two strains of *N. barkeri*, the comparative transcriptome and proteome were analyzed. We speculated that genes related to heat acclimation might be associated with heat shock protein, energy metabolism and immune system, which may have a different expression pattern from sudden heat stimulation. Response to heat tolerance is a complex process and our study could be conducive to identify the mechanisms and molecular pathways that are responsible for the capacity of acclimated predatory mite *N. barkeri* to maintain metabolic homeostasis and survive under thermal stress and heat acclimation.

## Materials and Methods

### Mites Rearing and Sampling

The CS and HTAS of *N. barkeri* were kept at a 25 ± 1°C and 35 ± 1°C, respectively with 70–80% RH and L: D = 14:10 h photoperiod in a climate-controlled room (Zhang G. H. et al., 2018). The CS mites were originally obtained from the Institute of Plant Protection, Chinese Academy of Agricultural Sciences in 2010. The HTAS mites had been heat acclimated for at least 300 generations at 35 ± 1°C and heat hardened at 42°C for 70 times (every 15–25 days) before our study. Both colonies were separately maintained on plastic non-transparent sheets resting on wet cotton and fed daily with the two-spotted spider mite (*Tetranychus urticae*) of different life stages. Five hundred newly emerged females (1–2-day-old) from each colony were collected into a 1.5-mL centrifuge tube respectively, and stored as a replicate sample at –80°C for total RNA or protein preparation.

### Preparation of cDNA and Transcriptome Sequencing

For each strain, three replicate samples were prepared for the total RNA isolation using Trizol Reagent (Invitrogen, CA, United States) according to the manufacturer’s protocol. The integrity and quantity of RNA were determined using the Agilent 2100 Bioanalyzer (Agilent Technologies, CA, United States).

Library preparations were sequenced on the BGIseq-500 platform (BGI, Shenzhen, China) (Zhu et al., 2018). The library was amplified by phi29 to make DNA nanoball (DNB) which has >300 copies of a molecular. DNBs were load into the patterned nanoarray, and the single end 50 bases reads were produced in the way of sequenced via synthesis. The DNB patterned array technology not only provides sequencing accuracy but also increases chip utilization and sample density.

### Annotation and Identification of Differentially Expressed Genes

To identify the differentially expressed genes from *N. barkeri* that are regulated by heat acclimation at a transcriptional level, a FDR (false discovery rate) ≤ 0.001 and an absolute value of log_2_ (fold-change HTAS/CS) ≥ 1 were used as the thresholds to screen DEGs (differentially expressed genes) (Love et al., 2014). For functional annotation, distinct sequences were searched via BLAST against the NCBI NR database with a cut-off *E*-value of 10^–5^. Blast2GO^1^ was used to assign Gene Ontology terms (GO^2^), while the database resource of Kyoto Encyclopedia of Genes and Genomes (KEGG)^3^ which integrates genomic, chemical, and systemic functional information was adopted to annotate molecular networks (pathways). The transcriptome raw reads were deposited in the Sequence Read Archive (SRA) bioproject number PRJNA492849.

### Protein Preparation, iTRAQ Labeling and SCX Fractionation

Six samples were grounded into powder in liquid nitrogen and extracted with lysis buffer (8 M Urea, 40 mM Tris-HCl, 2 mM EDTA (Ethylene diamine tetraacetic acid) (E1170, Solarbio, China) and 10 mM DTT (Dithiothreitol) (D1070, Solarbio, China) pH 8.5, respectively. The mixtures were placed into a Tissue Lyser for 2 min at 50 HZ to release proteins. After centrifugation with 25,000 *g* at 4°C for 20 min, the supernatant was transferred into another new tube. To reduce disulfide bonds, 10 mM DTT was added and incubated at 56°C for 1 h. Then, the mixtures were alkylated by 55 mM IAA (Iodoacetamide) (I8010, Solarbio, China) in the dark at room temperature for 45 min. Following centrifugation (25,000 *g*, 4°C, 20 min), the supernatant containing proteins was quantified by Bradford assay (P0006, Beyotime, China).

The protein solution (100 μg) with 8 M urea was diluted four times with 100 mM TEAB (Triethylammonium Bicarbonate) (T7951, Solarbio, China)and digested with Trypsin Gold (Promega, Madison, WI, United States) with the ratio of protein: trypsin = 40:1 at 37°C overnight. After trypsin digestion, peptides were desalted with a Strata X column (Phenomenex, United States) and vacuum-dried according to the manufacturer’s protocol for 8plex iTRAQ reagent (Applied Biosystems, Foster City, CA, United States). Samples were labeled with iTRAQ reagent. The peptides were dissolved in 30 μL 0.5 M TEAB with vortex. After the iTRAQ labeling reagents were recovered to ambient temperature, they were transferred and combined with proper samples. The labeled peptides with different reagents were combined and desalted with the Strata X column (Phenomenex, UCA) and vacuum-dried. The peptides were separated on a Shimadzu LC-20AB HPLC Pump system coupled with a high pH RP column. The peptides were reconstituted with buffer A1 (5% acetonitrile, 95% H_2_O, adjust pH to 9.8 with ammonia) to 2 mL and loaded onto a column containing 5 μm particles (Phenomenex, United States). The peptides were separated at a flow rate of 1 mL/min with a gradient of 5% buffer B1 (5% H_2_O, 95% acetonitrile, adjust pH to 9.8 with ammonia) for 10 min, 5–35% buffer B1 for 40 min, 35–95% buffer B1 for 1 min. The system was then maintained in 95% buffer B1 for 3 min and decreased to 5% within 1 min before equilibrating with 5% buffer B1 for 10 min. Then, elution was monitored by measuring absorbance at 214 nm, and the fractions were collected every 1 min. The eluted peptides were pooled as 20 fractions and vacuum-dried. Each fraction was resuspended in buffer A2 (2% acetonitrile and 0.1% formic acid in water) and centrifuged at 20,000 *g* for 10 min. The supernatant was loaded onto a C18 trap column 5 μL/min for 8 min using a LC-20AD nano-HPLC instrument (Shimadzu, Kyoto, Japan) by the auto-sampler. Then, the peptides were eluted from trap column and separated by an analytical C18 column (inner diameter 75 μm) packed in-house. The gradient was run at 300 nL/min starting from 8 to 35% of buffer B2 (2% H_2_O and 0.1% formic acid in acetonitrile) in 35 min, then going up to 60% in 5 min, then maintenance at 80% buffer B2 for 5 min, and finally returned to 5% in 0.1 min and equilibrated for 10 min.

### Mass Spectrometry (MS) Analysis

The peptides separated from nanoHPLC were subjected into the tandem mass spectrometry Q EXACTIVE (Thermo Fisher Scientific, San Jose, CA, United States) for DDA (data-dependent acquisition) detection by nano-electrospray ionization to achieve a better coverage and reliable statistics. Mass spectrometry data were acquired in peptide recognition mode using the 20 most abundant precursor ions from a survey scan of 350–1600 at a resolution of 70,000 in orbitrap. The target value (full MS target and MS2 target: 3e6 and 1e5) was determined by predictive Automatic Gain Control with dynamic exclusion for selected precursor ions 15 s, and under fill ratio 0.1%. The under fill ratio is the minimum percentage of the target value likely to be reached at the maximum fill time.

### iTRAQ Protein Identification and Quantification

The raw MS/MS data was converted into Mascot Generic Format (MGF) by Thermo scientific tool Proteome Discoverer (Thermo Fisher Scientific, United States), and the exported MGF files were searched using Mascot version 2.3.02 (Matrix Science, London, United Kingdom). The mass spectrometry proteomics data have been deposited with the ProteomeXchange Consortium via the PRIDE partner repository with the data set identifier PXD011379. For protein identification, the peptide mass tolerance was 20 ppm and the fragment mass tolerance was 0.05 Da. Only peptides with a score above the probability-based Mascot identity threshold at the 95% confidence interval were counted as identified. To identify a protein, the global FDR must be less than 1% and each protein involved at least one unique peptide. For protein quantization by an automated software called IQuant for quantitatively analyzing the labeled peptides with isobaric tags, a protein must contain at least two unique peptides.

### GO and Pathway Enrichment Analysis

Functional annotation of proteins identified in *N. barkeri* samples was carried out using Blast2GO which assigns gene ontology through BLAST searches against protein databases. The differentially expressed proteins were defined to be significantly regulated if the *P*-value is less than 0.05. The hyper geometric test was used to get the target GO terms in the GO enrichment analysis. The formula is as follows:

$$P=1-\sum_{j=0}^{M-1}\frac{\left(\begin{array}{c} M\\ j \end{array}\right)\,\left(\begin{array}{c} N-M\\ n-j \end{array}\right)}{\left(\begin{array}{c} N\\ n \end{array}\right)}$$

Where *N* is the number of all identified proteins which matched to GO terms; *n* is the number of differentially expressed proteins; *M* is the number of proteins which matched a certain GO term and *m* is the number of differentially expressed proteins which matched a certain GO term. If the *P*-value is less than 0.05, the GO term is significantly enriched in differentially expressed proteins. All identified proteins were mapped to a pathway in the Kyoto Encyclopedia of Genes and Genomes (KEGG) database. Significantly enriched pathways containing differentially expressed proteins were identified using the same formula as in GO analysis.

### Correlation Between Protein and mRNA Expression

To obtain the correlation between transcriptomic and proteomic database, the cutoff values were chosen to select the subsets of genes and proteins with distinctive expression signals. For each protein, we queried the RNA-seq data for expression patterns of matching transcripts (*P*-value < 0.05).

### qRT-PCR Analysis

The genes selected according to the differentially expressed genes (DEGs) and differentially expressed proteins (DEPs) were investigated by qRT-PCR at the transcriptional level. The housekeeping gene β*-actin* was used as a reference gene (Wang C. et al., 2018) for normalization purposes and the primers used in this study were listed in Supplementary Table S1. One μg of total RNA was used to synthesize the first-strand cDNA using the PrimeScript^TM^ RT reagent Kit (TaKaRa, Dalian, China). The qRT-PCR was determined with a Qtower3 System (Analytik Jena, Germany) using GoTaq qPCR Master Mix (Promega, Madison, WI, United States) according to the following thermal program: 1 cycle of 95°C for 2 min, followed by 40 cycles of 95°C for 15 s and 60°C for 30 s. The relative expression levels were calculated using the 2^–ΔΔ*Ct*^ method (Livak and Schmittgen, 2001). All data was given in terms of relative mRNA expression as mean ± SE.

## Results

### RNA-seq and iTRAQ Data Analysis

Approximately a total of 66.14 Gb transcriptome data and 39,623 unigenes were obtained from six samples of *N. barkeri*. The total length, average length, N_50_, and GC content of unigenes were 56,432,972 bp, 1,412 bp, 2,592 bp and 49.12%, respectively (Supplementary Table S2). Analysis found that there were 23,128 (58.37%), 9,313 (23.50%), 17,338 (43.76%), 17,035 (42.99%), 17,140 (43.26%), 3,853 (9.72%), and 17,475 (44.10%) unigenes mapped to the NR, NT, Swiss-Prot, euKaryotic Ortholog Groups (KOG), Kyoto Encyclopedia of Genes and Genomes (KEGG), Gene Ontology (GO) and InterPro databases, respectively. The *N. barkeri* sequences had their best hits with sequences from predatory mite, *Metaseiulus occidentalis* (81.4%), followed by *Ixodes scapularis* (1.4%) and *Limulus pjlolyphemus* (1.2%) (Supplementary Figure S1).

The total number of sequences identified by mass spectrometry of *N. barkeri* proteomes was 336,503, which represented 92,671 peptide spectra and 24,811 distinct peptides (Supplementary Table S3). Among the 24,811 identified peptides, only 20% (5,082) were assigned to a putative protein by homology search against the transcriptome database, leaving approximately 80% (19,729) of the peptides unidentified.

### Differentially Expressed Genes (DEGs) Between CS and HTAS

**FIGURE 1:**
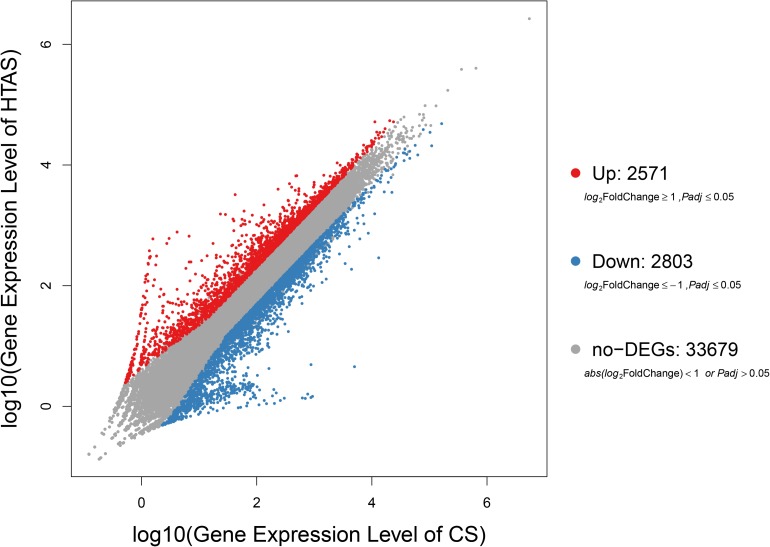
Expression levels in HTAS vs. CS female adults. The variability pattern was displayed by volcano plot. Red points, blue points, and gray points represent up-regulated genes, down-regulated genes, and non-differentially expressed genes, respectively. The horizontal coordinate indicates expression level in CS female adults, while the vertical coordinate indicates expression level in HTAS female adults.

After heat acclimation, a total of 2,571 and 2,803 up- and down-regulated transcripts were differentially expressed in HTAS, respectively (FDR ≤ 0.001 and log_2_Ratio ≥ 1) (Figure 1). The top ten up-regulated genes that respond to heat acclimation were as follows: fructose-2,6-bisphosphatase, stromal interaction molecule, heat shock factor protein, phosphatidylinositol-binding clathrin assembly protein, AH receptor-interacting protein, calcium-binding protein, microtubule affinity-regulating kinase, mucolipin, zinc finger protein, single-stranded DNA-binding protein and DNA replication licensing factor (Supplementary Table S4).

**FIGURE 2:**
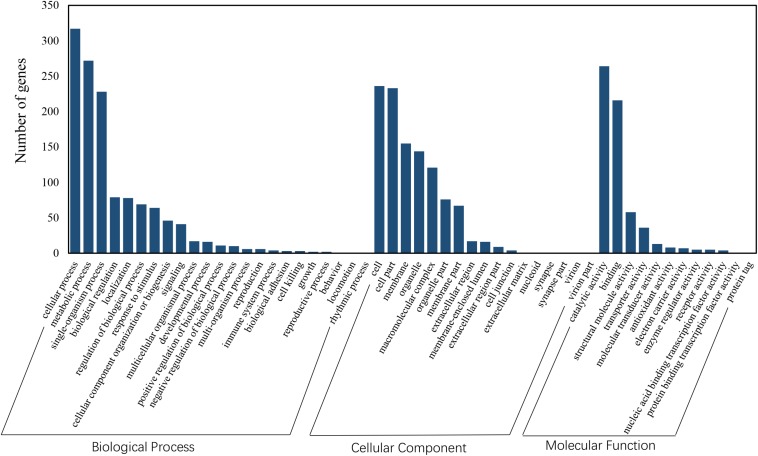
GO enrichment analysis revealed the biological processes most associated with detected DEGs. Based on the GO results, “cellular process,” “metabolic process,” “membrane” and “catalytic activity” were the most enriched GO terms under heat acclimation.

**FIGURE 3:**
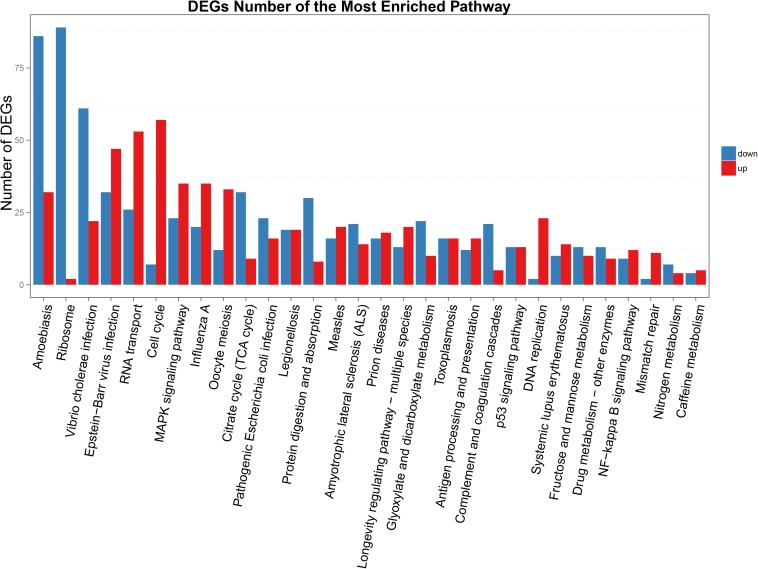
KEGG enrichment analyses of DEGs. The *X*-axis indicates KEGG pathway entries, and the *Y*-axis indicates the number of the DEGs. The red bar indicates up-regulated genes, and the blue bar indicates down-regulated genes.

**FIGURE 4:**
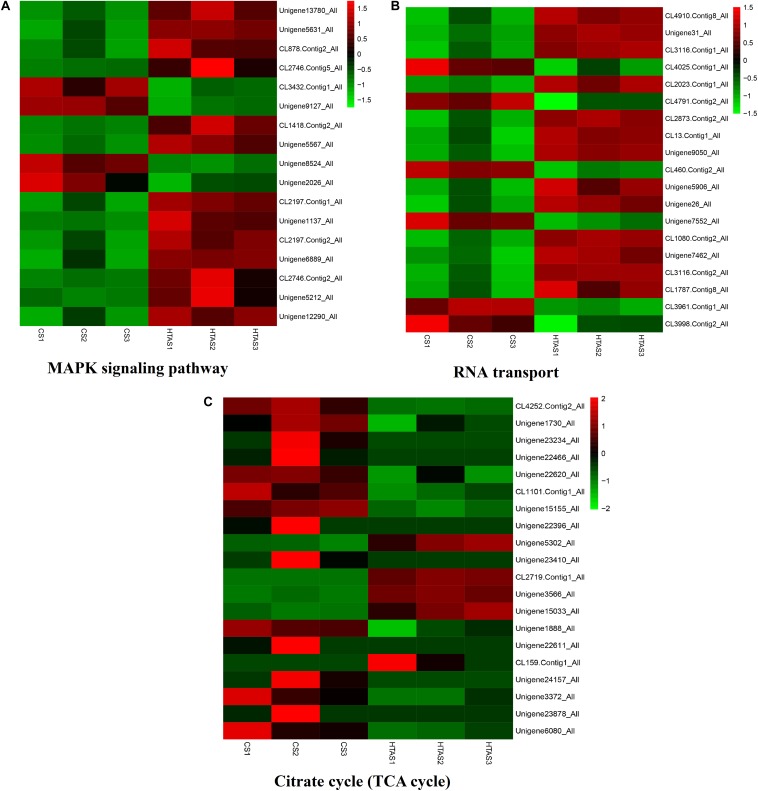
Heat map of the expression levels of different pathways. The expression levels of some genes in “MAPK signaling pathway,” “RNA transport” and “Citrate cycle (TCA cycle)” were shown in **(A–C)**, respectively. The color scale is shown at the upper right, which denotes FPKM value from the lowest (green) to the highest (red).

Functional annotation showed that 2,979 transcripts were differentially expressed between two strains in GO classification (≥2-fold change, FDR ≤ 0.001). These 2,979 differentially expressed genes were assigned to 53 GO terms (Figure 2) and classified into three categories including biological process, cellular component and molecular function, and mostly responses to the cellular process, metabolic process, single-organism process, cell, cell part, membrane, organelle, binding and catalytic activity. In KEGG analysis, 4,979 DEGs were mapped to 299 pathways. Among the DEGs, many pathways including ribosome, RNA transport, MAPK signaling pathway, citrate cycle, glyoxylate and dicarboxylate metabolism and fructose mannose metabolism were enriched (Figure 3). More specifically, we found mostly up-regulated genes were linked to MAPK signaling pathway, RNA transport and cell cycle (Table 1 and Figure 4). It is well known that genes encoding the heat shock proteins and the protective enzymes play a vital role in thermal tolerance in *N. barkeri* and other insects. In our present study, we found 9 out of 15 HSPs up-regulated in HTAS. However, the protective enzymes including glutathione S-transferase, superoxide dismutase, peroxidase and cytochrome P450 displayed down-regulated expression in HTAS strain (Table 2).

**TABLE 1:** Significantly enriched KEGG pathways in the transcriptome.

	Pathway	DEGs genes with pathway annotation (2843)	All genes with pathway annotation (17140)	*P-value*
1	Amoebiasis	118(4.15%)	455(2.65%)	1.99e-07
2	Ribosome	91(3.2%)	301(1.76%)	2.33e-09
3	*Vibrio cholerae* infection	83(2.92%)	369(2.15%)	0.00181705
4	Epstein-Barr virus infection	79(2.78%)	79(2.78%)	0.04908091
5	RNA transport	79(2.78%)	388(2.26%)	0.02771446
6	Cell cycle	64(2.25%)	207(1.21%)	2.18e-07
7	MAPK signaling pathway	58(2.04%)	290(1.69%)	0.06977517
8	Influenza A	55(1.93%)	268(1.56%)	0.05101354
9	Oocyte meiosis	45(1.58%)	214(1.25%)	0.05111887
10	Citrate cycle (TCA cycle)	41(1.44%)	183(1.07%)	0.02444778
11	Pathogenic Escherichia coli infection	39(1.37%)	187(1.09%)	0.07256013
12	Legionellosis	38(1.34%)	98(0.57%)	1.18e-07
13	Protein digestion and absorption	38(1.34%)	175(1.02%)	0.04526843
14	Measles	36(1.27%)	145(0.85%)	0.00703467
15	Amyotrophic lateral sclerosis (ALS)	35(1.23%)	154(0.9%)	0.02919038
16	Prion diseases	34(1.2%)	102(0.6%)	2.59e-05
17	Longevity regulating pathway-multiple species	33(1.16%)	145(0.85%)	0.03282006
18	Glyoxylate and dicarboxylate metabolism	32(1.13%)	140(0.82%)	0.0332671
19	Toxoplasmosis	32(1.13%)	142(0.83%)	0.03976848
20	Antigen processing and presentation	28(0.98%)	124(0.72%)	0.05059105
21	Complement and coagulation cascades	26(0.91%)	91(0.53%)	0.002884705
22	p53 signaling pathway	26(0.91%)	79(0.46%)	0.000279262
23	DNA replication	25(0.88%)	66(0.39%)	2.66e-05
24	Systemic lupus erythematosus	24(0.84%)	91(0.53%)	0.01185341
25	Fructose mannose metabolism	23(0.81%)	67(0.39%)	0.000309300
26	Drug metabolism-other enzymes	22(0.77%)	95(0.55%)	0.06053302
27	NF-kappa B signaling pathway	21(0.74%)	79(0.46%)	0.01628801
28	Mismatch repair	13(0.46%)	40(0.23%)	0.0100839
29	Nitrogen metabolism	11(0.39%)	33(0.19%)	0.0141972
30	Caffeine metabolism	9(0.32%)	27(0.16%)	0.0255240

**TABLE 2:** Differentially expressed genes within protective enzymes in two strains of *Neoseiulus barkeri.*

Gene	Gene ID	log2Fold Change (HTAS/CS)	Up/down regulation	*P-value*
Heat shock protein (HSP)	CL1418.Contig2_All	2.23699	Up	1.71E-37
	CL1418.Contig7_All	1.818885	Up	0.000859
	Unigene13412_All	1.361364	Up	0.000449
	Unigene7597_All	0.932925	Up	2.25E-07
	Unigene2581_All	0.854068	Up	1.49E-09
	Unigene21773_All	0.813447	Up	0.291441
	Unigene5806_All	0.772668	Up	5.01E-05
	Unigene4285_All	0.721268	Up	5.13E-06
	Unigene4291_All	0.64647	Up	0.000471
	Unigene22585_All	–1.88864	Down	0.0348
	Unigene23049_All	–2.06312	Down	0.021636
	Unigene23078_All	–2.35964	Down	0.008732
	CL2949.Contig2_All	–2.36266	Down	0.000899
	Unigene23749_All	–3.32123	Down	0.000149
	Unigene8220_All	–4.17255	Down	7.78E-07
Glutathione S-transferase (GST)	CL743.Contig4_All	2.661392	Up	0.002142
	CL508.Contig2_All	0.92927	Up	2.09E-08
	Unigene15461_All	0.900417	Up	1.59E-05
	CL3859.Contig2_All	0.813862	Up	0.291261
	CL2523.Contig2_All	0.802781	Up	0.014868
	Unigene7489_All	0.779944	Up	0.000137
	Unigene10826_All	0.515165	Up	0.000254
	Unigene8976_All	–0.58339	Down	0.018981
	CL2761.Contig2_All	–0.60901	Down	0.288457
	Unigene9195_All	–0.68444	Down	0.188359
	CL570.Contig1_All	–0.74015	Down	0.002268
	CL508.Contig4_All	–0.76566	Down	0.001365
	CL3265.Contig2_All	–0.77436	Down	5.19E-05
	CL743.Contig2_All	–0.88445	Down	0.005364
	Unigene7963_All	–0.99641	Down	0.257387
	CL2872.Contig2_All	–1.09984	Down	0.001081
Catalase	Unigene21196_All	0.813447	Up	0.291441
	CL3874.Contig1_All	–0.57308	Down	0.011095
	Unigene23703_All	–1.15575	Down	0.164561
	Unigene4824_All	–2.1239	Down	0.018363
Transient receptor potential cation channel (TRP)	CL2337.Contig2_All	2.088689	Up	0.00214
	CL4696.Contig2_All	0.622832	Up	5.72E-08
	Unigene10192_All	–0.51597	Down	0.004607
	Unigene8439_All	–0.59497	Down	0.010641
	Unigene6812_All	–0.63064	Down	0.025992
	Unigene15355_All	–0.66279	Down	0.294369
	Unigene15390_All	–0.75842	Down	0.035843
Superoxide dismutase (SOD)	Unigene21101_All	0.645468	Up	0.370641
	Unigene5500_All	0.590076	Up	0.070346
	Unigene22666_All	–0.92129	Down	0.248012
	Unigene24132_All	–2.18431	Down	0.015332
	Unigene23272_All	–3.32983	Down	0.000175
Peroxidase (POD)	CL1116.Contig4_All	–0.50819	Down	0.073818
	CL1116.Contig5_All	–0.80407	Down	0.008566
	CL1116.Contig2_All	–0.96238	Down	0.138795
	Unigene5513_All	–1.07244	Down	0.141829
	CL1116.Contig7_All	–1.28722	Down	0.077014
	CL1116.Contig3_All	–2.03096	Down	1.65E-21
Thioredoxin	Unigene13561_All	1.060005	Up	6.04E-07
	Unigene12250_All	0.9967	Up	1.77E-07
	Unigene14885_All	0.764948	Up	0.014423
	Unigene12285_All	0.673263	Up	1.61E-05
	Unigene7389_All	–1.15467	Down	1.66E-06
	Unigene23973_All	–1.32072	Down	0.121056
	Unigene22624_All	–3.56412	Down	4.44E-05
Cytochrome P450	CL643.Contig3_All	2.55122	Up	2.11E-23
	CL1009.Contig2_All	2.264515	Up	0.010601
	CL1009.Contig1_All	1.53405	Up	9.66E-11
	Unigene15379_All	1.264204	Up	2.89E-06
	CL405.Contig7_All	–1.10594	Down	0.000698
	CL3961.Contig1_All	–1.13247	Down	1.70E-07
	CL3199.Contig1_All	–1.33159	Down	0.009436
	CL238.Contig5_All	–1.48081	Down	3.65E-08
	CL1691.Contig1_All	–1.58524	Down	3.06E-15
	CL2671.Contig2_All	–1.64152	Down	0.000239
	CL2671.Contig1_All	–1.66389	Down	0.000536
	CL1617.Contig1_All	–1.70179	Down	2.49E-06
	CL2334.Contig1_All	–1.774	Down	3.12E-06
	CL405.Contig6_All	–1.88094	Down	2.97E-08
	CL1691.Contig3_All	–1.92396	Down	4.64E-18
	CL4535.Contig2_All	–2.37904	Down	0.008156
	CL906.Contig2_All	–2.53014	Down	3.60E-11

### Heat Tolerance-Dependent Protein Expression in Two Strains of N. barkeri

At proteome, a total of 500 differentially expressed proteins were identified in HTAS in comparison with CS, with 225 proteins up-regulated and 275 down-regulated after heat acclimation (Supplementary Table S3). Isocitrate dehydrogenase, an essential enzyme in the mitochondrial antioxidant system (Choi et al., 2018), was up-regulated by 3.16-fold in HTAS. Other up-regulated proteins were also identified in HTAS strain, including Chymotrypsin B (2.45-fold) which displays positive regulation of apoptotic process (Orlowski, 1999); methyltransferase (2.14-fold) involves in environmentally induced epigenetic modification mechanism of DNA methylation (Bird, 2002); serpin B10 (1.71-fold) an immune-related protein, plays a role in the regulation of protease activities during hematopoiesis and apoptosis (Schleef and Chuang, 2000), as well as cytochrome P450 (1.64-fold) which is ubiquitous heme-containing monooxygenases involve in a number of vital processes including detoxication and fatty acid metabolism (Nebert and Gonzalez, 1987). Proteins down-regulated in HTAS included maleylacetoacetate isomerase (0.5-fold) which catalyzes the GSH-dependent isomerization of maleylacetoacetate and play vital role in tyrosine metabolism (Kim et al., 2011); PHD finger (0.52-fold) which performs essential roles in the regulation of histone methylation and is an essential factor for epigenetic regulation and genome maintenance (Liu et al., 2018); calcineurin-like phosphoesterase domain-containing protein 1 (*CPPED1*) (0.53-fold) which improves adipocyte glucose metabolism when downregulation of its expression (Vaittinen et al., 2013); Ras-like GTP-binding protein family (0.53-fold) which are components of signaling pathways that link extracellular signals via trans-membrane receptors to cytoplasm and touch on virtually every aspect of cell biology including growth, differentiation, morphogenesis, cell division and motility and cytokinesis (Wang et al., 2017); vitellogenin (0.63-fold) which belongs to a family of lipid transport proteins and is associated with reproduction (Sappington and Raikhel, 1998) and glutathione S-transferase (0.64-fold) which is involved in detoxification and antioxidant defense machinery protects against oxidative stress (Chasseaud, 1979; Gill and Tuteja, 2010) (Supplementary Tables S5, S6).

**FIGURE 5:**
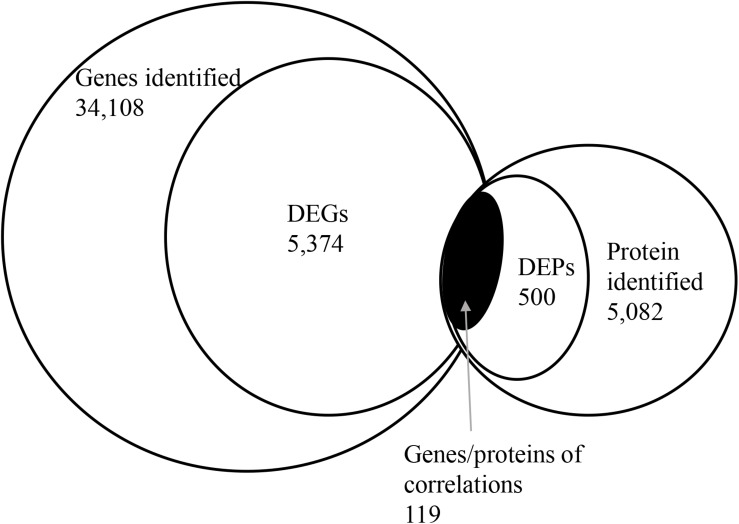
Correlation between differentially expressed proteins and genes. The numerical value in each circle represents the quantity of genes or proteins, including identified genes and proteins, and genes or proteins related to heat acclimation respectively, and genes/proteins related to heat acclimation together.

**FIGURE 6:**
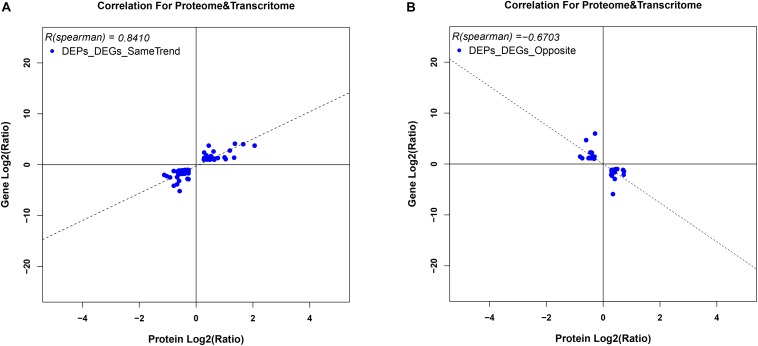
Variation in trends between DEGs and DEPs from the same locus. **(A)** represents DEGs and DEPs with the same trend, while **(B)** represents DEGs and DEPs with opposite trends. Scatter plots illustrate the distribution of differentially expressed proteins and related genes. The Pearson correlation coefficient between protein and mRNA expression profiles is shown in the upper left corner of the plot.

To correlate protein and mRNA expression profiles, genes with both quantifiable mRNA and protein data were extracted and compared with the annotated RNA-seq libraries. Correlation between DEGs and DEPs showed 119 genes/proteins related to heat acclimation (Figure 5). Supplementary Tables S5, S6 represented the correlation between mRNA and protein, and the correlation coefficients between the protein and gene expression profiles of the same trend and opposite trend were 0.8410 and -0.6703, respectively (Figure 6 and Supplementary Tables S7, S8).

### Gene Ontology and Pathway Enriched Analysis

**FIGURE 7:**
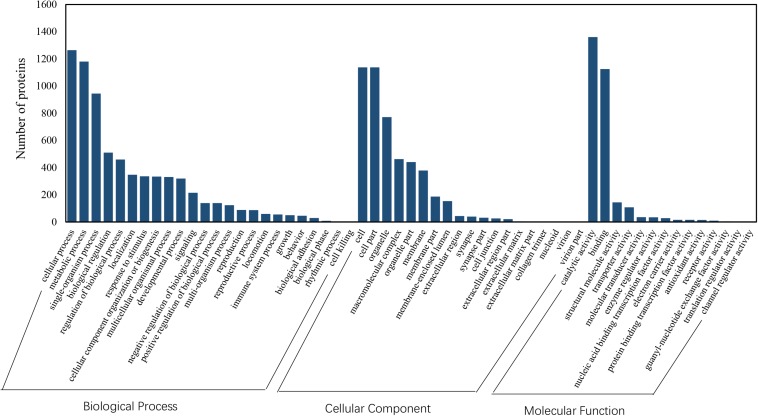
GO classification of proteins identified in *N. barkeri* female adults. The proteins are grouped into three hierarchically structured GO terms, biological process, cellular component, and molecular function.

Among the 5082 proteins, 2415 were sub-categorized into 57 hierarchically structured GO classes, including 24 biological process, 19 cellular component, and 14 molecular function, which mostly response to cellular process (52.3%), metabolic process (48.8%), cell part (47.1%), and catalytic activity (56.3%) (Figure 7). Three hundred and forty eight differentially expressed proteins were allocated to the reference pathways in KEGG (Table 3). As a result, 23 pathways were enriched (*P*-value ≤ 0.05, Table 3). Correlation of the enriched pathways for DEGs and DEPs showed there were four enriched pathways related to heat acclimation including amoebiasis, glyoxylate and dicarboxylate metabolism, citrate cycle (TCA cycle) and protein digestion and absorption (Tables 1, 3).

**TABLE 3:** Significantly enriched KEGG pathways in the proteome.

	Pathway	Diff Proteins with pathway annotation (348)	All Proteins with pathway annotation (3998)	*P-value*
1	Lysosome	28(8.05%)	145(3.63%)	3.522485e-05
2	Proteoglycans in cancer	21(6.03%)	127(3.18%)	0.002744244
3	Fluid shear stress and atherosclerosis	19(5.46%)	105(2.63%)	0.001481644
4	Apoptosis	18(5.17%)	90(2.25%)	0.0005803898
5	Autophagy - animal	16(4.6%)	94(2.35%)	0.006410966
6	Platelet activation	12(3.45%)	76(1.9%)	0.02963325
7	Leukocyte transendothelial migration	12(3.45%)	50(1.25%)	0.0009059034
8	Ras signaling pathway	11(3.16%)	68(1.7%)	0.0311076
9	Tuberculosis	11(3.16%)	63(1.58%)	0.01844893
10	Amoebiasis	11(3.16%)	57(1.43%)	0.008844626
11	Antigen processing and presentation	11(3.16%)	55(1.38%)	0.006712193
12	Pancreatic secretion	11(3.16%)	53(1.33%)	0.005005168
13	Neurotrophin signaling pathway	11(3.16%)	50(1.25%)	0.00310777
14	Sphingolipid signaling pathway	10(2.87%)	62(1.55%)	0.03956137
15	Rheumatoid arthritis	10(2.87%)	55(1.38%)	0.01848339
16	Pathogenic *Escherichia coli* infection	9(2.59%)	40(1%)	0.006224044
17	Adherens junction	8(2.3%)	45(1.13%)	0.03776486
18	Arachidonic acid metabolism	8(2.3%)	40(1%)	0.01967871
19	VEGF signaling pathway	8(2.3%	31(0.78%)	0.00401926
20	Glyoxylate and dicarboxylate metabolism	7(2.01%)	41(1.03%)	0.06079678
21	Citrate cycle (TCA cycle)	7(2.01%)	40(1%)	0.04428566
22	Protein digestion and absorption	7(2.01%)	7(2.01%)	0.04428566
23	Steroid biosynthesis	4(1.15%)	12(0.3%)	0.01589752

### Validation of Data Through Real-Time PCR (qRT-PCR)

**FIGURE 8:**
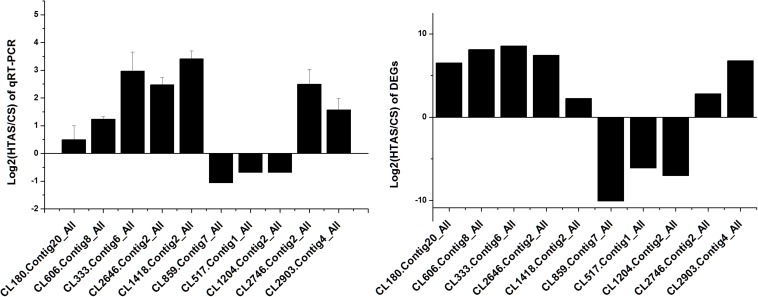
qRT-PCR analysis of the expression levels of 10 Unigenes. *X* axis represents different Unigenes. CL333.Contig6_All, fructose-2,6-bisphosphatase; CL2646.Contig2_All, calcium-binding protein; CL2903.Contig4_All, mucolipin; CL180.Contig20_All, single-stranded DNA-binding protein; CL606.Contig8_All, stromal interaction molecule; CL859.Contig7_All, DNA polymerase; CL517.Contig1_All, RING finger and SPRY domain-containing protein; CL1204.Contig2_All, long-chain fatty acid; CL1418.Contig2_All, HSP70 and CL2746.Contig2_All, HSP20. Y axis represents the relative expression levels of genes.

Thousands of genes showed significantly different expression levels in this study. As a result, 10 genes and 10 proteins were selected to confirm their expression levels through qRT-PCR. Expression patterns were validated among the ten annotated transcripts (CL333.Contig6_All, CL2646.Contig2_All, CL2903.Contig4_All, CL180.Contig20_All, CL606.Contig8_All, CL859.Contig7_All, CL517.Contig1_All, CL1204.Contig2_All, CL1418.Contig2_All and CL2746.Contig2_All). Two heat shock protein genes, *HSP70* (CL1418.Contig2_All) and *HSP20* (CL2746.Contig2_All), showed up-regulated expression. The changing trend of the qRT-PCR results was consistent with the DEG expression profiling (Figure 8).

## Discussion

Thermal acclimation can significantly alter the thermotolerance of invertebrates, such as *Tribolium castaneum* (Lü and Huo, 2018), *Drosophila melanogaster* (Colinet et al., 2013) and *Nilaparvata lugens* (Piyaphongkul et al., 2014). For example, high temperature acclimation (42°C) could significantly improve the heat tolerance of adult females of *T. castaneum* (Lü and Huo, 2018). Our recent study proved that a long-term high temperature adapted strain (HTAS) of *N. barkeri* (acclimated at 35°C) could withstand more severe heat stress, with a significantly lower mortality caused by heat stress than CS mites did (Zhang G. H. et al., 2018). This intraspecific variation on thermal susceptibility between HTAS and CS suggested *N. barkeri* might had been shaped by a differentially physiological strategy in response to temperature changes.

As expected, the physiological strategy of HTAS *N. barkeri* to heat acclimation was related to the expression of some unique genes and enriched pathways. In total, 5,374 DEGs (2,571 up-regulated and 2,803 down-regulated) has been identified in response to heat acclimation. The GO analysis results suggested that most of DEGs were associated with the metabolic reaction, enzyme catalysis and membrane process. Similar results were observed in *T. castaneum* in response to heat stress with the membrane, metabolic process, and catalytic activity were remarkably enriched (Lü and Huo, 2018). Many DEGs associated with “membrane” suggesting the cells related to heat stress mechanically were injured since the membrane is concerned with the requirement for repair promotion (Howard et al., 2011). Moreover, some important signaling pathways were involved in heat adaptation on *N. barkeri* adults, such as “Ribosome,” “Citrate cycle (TCA cycle),” “Cell cycle,” “Glyoxylate and dicarboxylate metabolism,” “RNA transport” and “MAPK signaling pathway,” were enriched in HTAS mites, indicating the importance of these pathways in forming heat tolerance. In insects, the innate immune system is a major effector response system (Gillespie et al., 1997). MAPK signaling pathway plays an important role in the immune processes of a variety of organisms, particularly with respect to the regulation of the innate immune response (Zhan et al., 2018). It was proved that MAPK signaling pathway was activated by high temperature to break pupal diapause in the flesh fly (Fujiwara and Denlinger, 2007) and involved in the responses of *Dendranthema morifolium* to low temperature (Lu et al., 2018). Surprisingly, the majority of DEGs within the MAPK signaling pathway were up-regulated in HTAS adults, suggesting these humoral immune-related pathways were activated in response to the heat stress acclimation (Chen et al., 2017; Zhang Y. et al., 2018). However, the metabolism of HTAS for heat acclimation was weak since the pathways such as glyoxylate and dicarboxylate metabolism and citrate cycle (TCA cycle) were enriched a majority of down-regulated DEGs (Figure 4). A similar reduction was found in *Glyphodes pyloalis* responding to heat tolerance (Liu et al., 2017). The suppression of metabolism reaction may reflect cellular homeostasis and an energy-saving mechanism to manage heat stress.

Heat shock proteins (HSPs), acting as highly conserved molecular chaperones, displayed essential roles in various cellular processes, including preventing protein aggregation and denaturation and maintaining protein homeostasis during periods of thermal stress (Iwama et al., 1999). Fifteen HSPs of *N. barkeri*, including HSP40, HSP60, HSP70, HSP90 and several small HSPs, were observed and most of them exhibited up-regulated expression in HTAS according to the transcriptome analysis. The preferential induction of HSPs related to a thermal challenge has been documented in many previous studies. In *N. lugens*, HSP70 is constitutively responsive to heat shock but down-regulated to cold tolerance, while remarkably up-regulated after insecticide exposure, which reveals that HSP70 is evolutionarily and functionally diversified and involved in responses to environmental stresses such as the resistance or tolerance (Lu et al., 2017). Moreover, HSP70 could inhibit JNK and Caspase-3 activation to regulate innate immune responses (Shukla et al., 2014) and HSP60 could activate the stress-activated protein kinases p38 to regulate Toll signaling pathway in innate immune cells (Vabulas et al., 2001). In our previous study, HSP70 and HSP90 of CS *N. barkeri* female adults were significantly more intense in high temperature at 40°C for just 1.5 h, which suggested HTAS was more tolerant under heat stress (Xu et al., 2018). Our transcriptome analysis showed the induced expression of four HSP70 may accelerate the refolding of damaged proteins and prevent protein aggregation during heat acclimation. Nine out of 15 HSPs of HTAS, including 1 HSP10, 1 HSP40, 1 HSP60, 4 HSP70, and 2 HSP90, displayed up-regulated expression related to heat acclimation which served as an evolutionarily conserved mechanism.

Arthropods, like other aerobic organisms, face a constant oxidative challenge from the production of ROS during cellular metabolism when exposed to heat stress. Peroxidase genes have been confirmed on potential roles in protecting the organism from the oxidative damage challenge under three environmental stresses (thermal, UV and pesticide) in *N. barkeri* (Tian et al., 2017). It has been proved that the HTAS *N. barkeri* exhibited lower fitness under UV-B stress compared with the CS *N. barkeri* due to the lower levels of activity for some antioxidant enzymes in HTAS *N. barkeri* (Tian et al., 2019). Indeed, in this study, many antioxidant-related genes, including superoxide dismutase (SOD), peroxidase (POD), catalase (CAT) and thioredoxin were significantly down-regulated in HTAS (Table 2), which suggests a conservative progress of energy-saving response to deal with the toxic substance accumulation in HTAS and the difference in fitness cost between the two strains. Similar results were observed in detoxication-related genes including glutathione S-transferase (GST) and cytochrome P450. The detoxification pathways identified in our study will provide new insights for the investigation of the molecular mechanisms of fitness cost under environmental stresses.

Using high-throughput MS-based proteomics, 500 DEPs were determined including 225 up-regulated DEPs and 275 down-regulated DEPs. These 348 differentially expressed proteins were allocated to the reference pathways in KEGG, where 23 pathways were enriched (Table 3). Sphingolipid signaling pathway has been proved to be involved in defensive mechanisms (Thuleau et al., 2013) which may be contributed to activating the defensive response of *N. barkeri* by heat stress. The activities of antigen process and presentation, related to immune response (Kelly and Trowsdale, 2018) were modulated by heat acclimation. The upregulation of immune-related genes including serpin B10 (1.71-fold) under heat acclimation further suggested that heat stress facilitated the components of immune defense.

Correlation analysis of differentially expressed proteins and genes displayed that a subset of genes and proteins were expressed with the same trend related to heat acclimation (Supplementary Table S7). These genes including HSP70 (CL1418.Contig6), HSP90 (Unigene15778), isocitrate dehydrogenase (CL4252.Contig1), vitellogenin (CL125.Contig1) and ATP-dependent DNA helicase (Unigene12306) were up-regulated, while cytochrome P450 (CL1691.Contig3), cuticle protein (CL1299.Contig2), choline dehydrogenase (CL854.Contig1), endochitinase (CL4004.Contig2) and many unknown and uncharacterized protein were among the genes down-regulated at both the transcriptional and translational levels. Cuticular lipids are important to the fitness of the insects and a major barrier to water loss. The cuticle protein was downregulated in HTAS which may imply a fitness cost of heat acclimation in desiccation situation (Huang et al., 2019). Physiologically similar phenotypes were identified in *D. melanogaster* which the proteomic analysis (2D-DIGE) showed the physiological changes of the acclimated *D. melanogaster* adults could drastically alter expressed proteins especially HSP70 (Colinet et al., 2013). The up-regulated gene isocitrate dehydrogenase as an essential enzyme catalyzes oxidative decarboxylation of isocitrate to α-ketoglutarate and CO_2_ with a concomitant reduction of NADP^+^ to NADPH (Wise et al., 2011). Studies have been confirmed that isocitrate dehydrogenases enzymes are involved in the thermal properties in the bacterium, *Colwellia maris* (Mouri and Takada, 2018). However, there are some genes, including lipoprotein receptor (Unigene8357), glutamine synthetase (CL2142.Contig1) and serine/threonine-protein kinase (CL3245.Contig2), were up-regulated at the translational level but down-regulated at the transcriptional level (Supplementary Table S8). This effect may contribute to differences between genes and proteins in the posttranscriptional regulatory mechanism (He and Klionsky, 2009). Four pathways including amoebiasis, glyoxylate and dicarboxylate metabolism, citrate cycle (TCA cycle) and protein digestion and absorption were observed enriched in both DEGs and DEPs in HTAS. Citrate cycle (TCA cycle) metabolism was enriched in plant (Ren et al., 2018; Wang J. et al., 2018) and thermophilic bacteria (Cordova et al., 2017) and yeast (Wang D. et al., 2018) to obtain more ATP production and more NADH, since detoxification of furfural or phenolic compounds is an energy-consuming process under heat stress which indicated resistance to high temperature of *N. barkeri* was a process of accelerating energy consumption.

In this study, comparative analysis between the CS and the HTAS *N. barkeri* with 6-years heat acclimation of *N. barkeri* was firstly performed at a transcriptomic and proteomic level. The results of the GO and KEGG pathway enrichment analyses of 5,374 identified DEGs indicated the DEGs related to metabolism and immunity were enriched after heat acclimation. In addition, the transcriptome results revealed a number of genes that were potentially relevant to HSPs, antioxidant, and detoxication in *N. barkeri*. A majority of antioxidant-related genes and detoxication-related genes were down-regulated in HTAS, which suggested a conservative progress of energy-saving response and the balance of fitness cost. Correlation of DEGs and DEPs showed four pathways related to metabolism and ATP production were enriched, indicating the importance of energy and metabolism at high temperature. Genes relevant to HSPs and ATP production were up-regulated at both the transcriptional and translational levels. Excellently thermal tolerance in HTAS *N. barkeri* was a result of long-term heat acclimations and heat hardenings which is different from the response to a sudden heat stimulus. Six years of laboratory heat acclimation is likely to have had a profound impact on the developmental physiology of *N. barkeri*. Our results contributed to the better knowledge of the complex physiological response mechanism of heat acclimation and provide insight into the heat tolerance of insects.

## Data Availability Statement

The datasets generated for this study can be found in the transcriptome raw reads were deposited in the Sequence Read Archive (SRA) bioproject number PRJNA492849. The mass spectrometry proteomics data have been deposited with the ProteomeXchange Consortium via the PRIDE partner repository with the data set identifier PXD011379.

## Ethics Statement

The research project was conducted on mite species that are not subjected to any specific ethical issue and legislation.

## Author Contributions

CT, YL, and HL conceived and designed the experiments. CT, YL, JH, and WC performed the experiments, analyzed the data, and drafted the manuscript. ZW and HL participated in manuscript drafted and modification. All authors read and approved the final manuscript.

## Conflict of Interest

The authors declare that the research was conducted in the absence of any commercial or financial relationships that could be construed as a potential conflict of interest.

## Footnotes

1 http://www.blast2go.org

2 http://www.geneontology.org

3 http://www.genome.jp/kegg

## Supplementary Material

FIGURE S1 Summary of iTRAQ metrics from *Neoseiulus barkeri* proteomes.

TABLE S1 Distribution of annotated species.

TABLE S2 Summary of RNA-seq metrics of Unigenes from *Neoseiulus barkeri* transcriptomes.

TABLE S3 Primers used in the RT-qPCR in this study.

TABLE S4 Up-regulated genes in HTAS of *Neoseiulus barkeri* transcriptomes.

TABLE S5 Up-regulated proteins in HTAS of *Neoseiulus barkeri* proteomes.

TABLE S6 Down-regulated proteins in HTAS of *Neoseiulus barkeri* proteomes.

TABLE S7 Correlation between mRNAs and proteins with the same trend.

TABLE S8 Correlation between mRNAs and proteins with the opposite trend.
