# Supplementary figures and images for: Comparative Transcriptome and Proteome Analysis of Heat Acclimation in Predatory Mite Neoseiulus barkeri
Source: Front Physiol. 2020 Apr 29;11:426. doi: 10.3389/fphys.2020.00426 (PMC7201100; doi:10.3389/fphys.2020.00426)

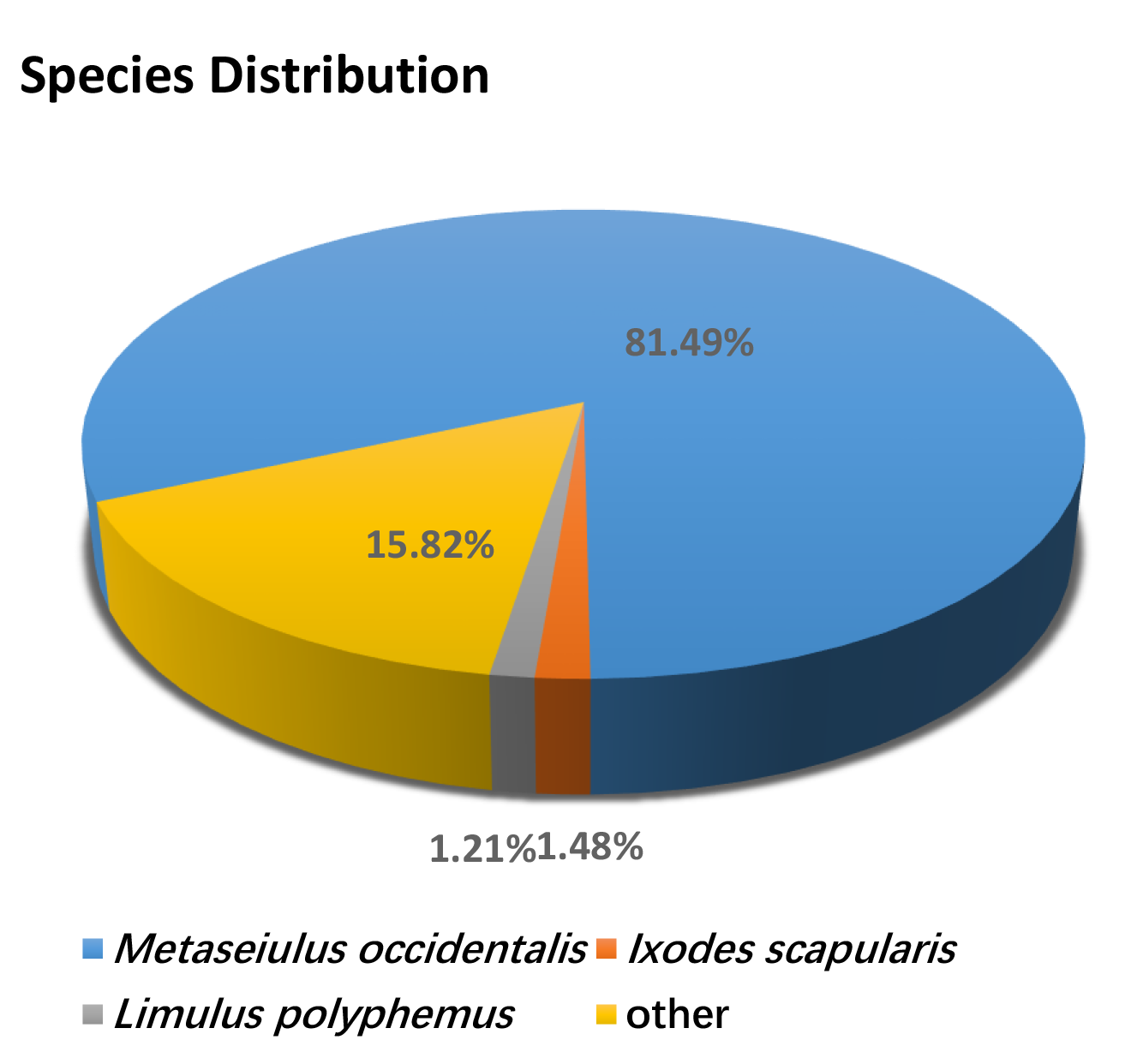

Supplement: FIGURE S1 — Summary of iTRAQ metrics from Neoseiulus barkeri proteomes. [file Image_1.TIF]
