# Supplementary material for: Comparative Transcriptome and Proteome Analysis of Heat Acclimation in Predatory Mite Neoseiulus barkeri
Source: Front Physiol. 2020 Apr 29;11:426. doi: 10.3389/fphys.2020.00426 (PMC7201100; doi:10.3389/fphys.2020.00426)
Supplement: TABLE S1 — Distribution of annotated species. [file Table_1.DOCX]

| Experiments | Primer | Sequence (5’ to 3’) |
| --- | --- | --- |
| RT-qPCR | CL180.Contig20_AllF | TAACGGACAATATGCGGTCA |
|  | CL180.Contig20_AllR | GCATTCCTCCAGGTCCACTA |
|  | CL606.Contig8_AllF | CTGACTCCTCAGGCTTGTCC |
|  | CL606.Contig8_AllR | CTTGTTTCCGTTGGTGTCCT |
|  | CL333.Contig6_AllF | TTGTGACGACCCGAACATTA |
|  | CL333.Contig6_AllR | TTCGGACTCGTCGAGACTTT |
|  | CL2646.Contig2_AllF | TATTTCAAACGCCGAGAACC |
|  | CL2646.Contig2_AllR | CTTCGAAAGGAAATCCACCA |
|  | CL1418.Contig2_AllF | AACAAGGCAACAGGACAACC |
|  | CL1418.Contig2_AllR | GGCAACTTTGAACGACCAAT |
|  | CL859.Contig7_AllF | CGAGCTACTTGGACCAGAGG |
|  | CL859.Contig7_AllR | ATCGAATTTTCTGGGCATTG |
|  | CL517.Contig1_AllF | CGGGATACTCAGGTGATGCT |
|  | CL517.Contig1_AllR | TTTCGTCGACCCACTCTTCT |
|  | CL1204.Contig2_AllF | AAAGCCCTAGAGGCGTTAGC |
|  | CL1204.Contig2_AllR | GAATTTCGTCGGAATCTCCA |
|  | CL2746.Contig2_AllF | GTCAACTGGTTCGACGATCA |
|  | CL2746.Contig2_AllR | TGGTTCTTGTTCGGGTTTTC |
|  | CL2903.Contig4_AllF | GACTTTCTGACCGGCTTGAG |
|  | CL2903.Contig4_AllR | CGAGCGTTTCAATGTCAGAA |
|  | β-actin-F | TACGACCAGAAGCGTACAGC |
|  | β-actin-R | CCAACCGTGAAAAGATGACC |

Table S1 Primers used in the RT-qPCR in this study
