# Supplementary material for: Comparative Transcriptome and Proteome Analysis of Heat Acclimation in Predatory Mite Neoseiulus barkeri
Source: Front Physiol. 2020 Apr 29;11:426. doi: 10.3389/fphys.2020.00426 (PMC7201100; doi:10.3389/fphys.2020.00426)
Supplement: TABLE S2 — Summary of RNA-seq metrics of Unigenes from Neoseiulus barkeri transcriptomes. [file Table_2.DOCX]

Table S2 Summary of RNA-seq metrics of Unigenes from [*Neoseiulus barkeri*](javascript:;) transcriptomes**.**

| **Metric** | **CS** | | **HTAS** | **All-Unigene** | |
| --- | --- | --- | --- | --- | --- |
| Clean Reads (Mb) | 109.9 | | 110.4 | 110.2 | |
| Unigene | 30,881 | | 29,266 | 39,623 | |
| Total length | 37,379,147 | | 37,379,147 | 56,432,972 | |
| Mean length | 1,212 | | 1,224 | 1,424 | |
| N50 | 2,132 | | 2,187 | 2,592 | |
| N70 | 1,373 | | 1,394 | 1,674 | |
| N90 | 501 | | 500 | 613 | |
| GC% | 49.15 | | 49.08 | 49.12 | |
| Annotation |  |  | | | 24,210 |
