# Supplementary material for: Comparative Transcriptome and Proteome Analysis of Heat Acclimation in Predatory Mite Neoseiulus barkeri
Source: Front Physiol. 2020 Apr 29;11:426. doi: 10.3389/fphys.2020.00426 (PMC7201100; doi:10.3389/fphys.2020.00426)
Supplement: TABLE S3 — Primers used in the RT-qPCR in this study. [file Table_3.DOCX]

#### Table S3 Summary of iTRAQ metrics form [*Neoseiulus barkeri*](javascript:;) proteomes.

| Metric | Number |
| --- | --- |
| Total spectra | 336,503 |
| Peptide spectra | 92,671 |
| Unique spectra | 81,205 |
| Distinct peptides | 24,811 |
| Matched protein | 5,082 |
| Differentially expressed protein | 500 |
