# Supplementary material for: Comparative Transcriptome and Proteome Analysis of Heat Acclimation in Predatory Mite Neoseiulus barkeri
Source: Front Physiol. 2020 Apr 29;11:426. doi: 10.3389/fphys.2020.00426 (PMC7201100; doi:10.3389/fphys.2020.00426)
Supplement: TABLE S4 — Up-regulated genes in HTAS of Neoseiulus barkeri transcriptomes. [file Table_4.DOCX]

Table S4 Up-regulated genes in HTAS of *Neoseiulus barkeri* transcriptomes.

| **Gene ID** | **Log2FoldChange**  **(HTAS/CS)** | **Up/Down-Regulation** | **P-value** | **NCBInr description** | **Pathway** |
| --- | --- | --- | --- | --- | --- |
| CL333.Contig6_All | 8.564393 | Up | 2.05E-39 | Fructose-2,6-bisphosphatase [*Metaseiulus occidentalis*] | Carbohydrate metabolism |
| CL606.Contig6_All | 8.115664 | Up | 3.11E-35 | Stromal interaction molecule homolog [*Metaseiulus occidentalis*] | Immune system |
| CL724.Contig5_All | 7.706229 | Up | 8.95E-28 | Heat shock factor protein-like [*Metaseiulus occidentalis*] | Longevity regulating |
| CL469.Contig2_All | 7.654133 | Up | 2.05E-45 | Phosphatidylinositol-binding clathrin assembly protein LAP-like [*Metaseiulus occidentalis*] | - |
| CL3340.Contig3_All | 7.497937 | Up | 1.49E-28 | AH receptor-interacting protein-like [*Metaseiulus occidentalis*] | - |
| CL2646.Contig2_All | 7.429433 | Up | 9.88E-28 | Calcium-binding protein 39-like [*Metaseiulus occidentalis*] | Signal transduction |
| CL198.Contig8_All | 6.971446 | Up | 9.92E-22 | Microtubule affinity-regulating kinase [*Metaseiulus occidentalis*] | Calcium signaling pathway |
| CL2903.Contig4_All | 6.766053 | Up | 4.78E-22 | Mucolipin, putative [*Ixodes scapularis*] | - |
| CL1122.Contig2_All | 6.63582 | Up | 4.34E-21 | Gastrula zinc finger protein like [*Octopus bimaculoides*] | - |
| CL180.Contig20_All | 6.522768 | Up | 1.59E-19 | Single-stranded DNA-binding protein 3-like [*Metaseiulus occidentalis*] | Signal transduction |
| CL2925.Contig2_All | 6.261793 | Up | 1.49E-31 | DNA replication licensing factor mcm5-A [*Metaseiulus occidentalis*] | Replication and repair |
| CL606.Contig8_All | 6.226196 | Up | 2.01E-16 | Stromal interaction molecule homolog [*Metaseiulus occidentalis*] | Immune system |
| CL578.Contig1_All | 6.071149 | Up | 2.20E-16 | PAX-interacting protein 1-like [*Metaseiulus occidentalis*] | - |
| CL595.Contig2_All | 6.038096 | Up | 2.57E-134 | Helicase POLQ-like [*Metaseiulus occidentalis*] | - |
| CL524.Contig1_All | 5.966441 | Up | 3.67E-16 | kynurenine 3-monooxygenase-like [Metaseiulus occidentalis] | Amino acid metabolism |
| CL4032.Contig1_All | 5.721552 | Up | 2.86E-97 | threonine-protein kinase BUB1 beta-like [Metaseiulus occidentalis] | Cell growth and death |
| CL613.Contig2_All | 5.403404 | Up | 3.30E-12 | DNA polymerase kappa-like [Metaseiulus occidentalis] | Replication and repair |
| CL161.Contig6_All | 5.400177 | Up | 1.09E-12 | Kynurenine--oxoglutarate transaminase 3, partial [Stegodyphus mimosarum] | Amino acid metabolism |
| Unigene3458_All | 5.203136 | Up | 1.09E-31 | Fructose-2,6-bisphosphatase-like [Metaseiulus occidentalis] | Carbohydrate metabolism |
