# Supplementary material for: Comparative Transcriptome and Proteome Analysis of Heat Acclimation in Predatory Mite Neoseiulus barkeri
Source: Front Physiol. 2020 Apr 29;11:426. doi: 10.3389/fphys.2020.00426 (PMC7201100; doi:10.3389/fphys.2020.00426)
Supplement: TABLE S5 — Up-regulated proteins in HTAS of Neoseiulus barkeri proteomes. [file Table_5.DOCX]

Table S5 Up-regulated proteins in HTAS of *Neoseiulus barkeri* proteomes.

| **Protein ID** | **Mean Ratio**  **HTAS/CS** | **Up/Down-Regulation** | **P-value** | **NCBInr Description** |
| --- | --- | --- | --- | --- |
| Gene.43257 | 5.55 | Up | 0.003882 | uUrease accessory protein ureG [*Metaseiulus occidentalis*] |
| Gene.9488 | 5.28 | Up | 0.000937 | Uncharacterized protein |
| Gene.16282 | 4.17 | Up | 0.001598 | Cuticle protein 14 [*Metaseiulus occidentalis*] |
| Gene.27007 | 3.16 | Up | 0.001206 | Isocitrate dehydrogenase [NADP] [*Metaseiulus occidentalis*] |
| Gene.7354 | 2.95 | Up | 0.02655 | uncharacterized protein [*Metaseiulus occidentalis*] |
| Gene.42169 | 2.63 | Up | 0.003966 | FAD-linked sulfhydryl oxidase ALR [*Metaseiulus occidentalis*] |
| Gene.30386 | 2.58 | Up | 0.008589 | Integrase core domain protein [*Trichinella spiralis*] |
| Gene.20610 | 2.45 | Up | 0.003536 | Chymotrypsin B [*Metaseiulus occidentalis*] |
| Gene.47618 | 2.23 | Up | 0.006024 | Uncharacterized protein [*Metaseiulus occidentalis*] |
| Gene.23031 | 2.14 | Up | 0.01464 | Methyltransferase-like protein [*Metaseiulus occidentalis*] |
| Gene.31399 | 2.11 | Up | 0.02706 | DNA-directed RNA polymerase III subunit RPC3 [*Metaseiulus occidentalis*] |
| Gene.45933 | 2.06 | Up | 0.01662 | ATP-dependent DNA helicase Q5 [*Metaseiulus occidentalis*] |
| Gene.18346 | 2 | Up | 0.007495 | Calcineurin-like phosphoesterase domain-containing protein [*Metaseiulus occidentalis*] |
| Gene.46065 | 1.97 | Up | 0.01879 | ADIPOR-like receptor [*Metaseiulus occidentalis*] |
| Gene.40109 | 1.9 | Up | 0.04652 | uncharacterized protein [*Metaseiulus occidentalis*] |
| Gene.47704 | 1.86 | Up | 0.02272 | RNA-directed DNA polymerase from mobile element jockey [*Metaseiulus occidentalis*] |
| Gene.31663 | 1.85 | Up | 0.02246 | Solute carrier family 22 member 5 [*Metaseiulus occidentalis*] |
| Gene.35408 | 1.85 | Up | 0.03298 | F-box/LRR-repeat protein 7 [*Metaseiulus occidentalis*] |
| Gene.16648 | 1.71 | Up | 0.007928 | Serpin B10 [*Metaseiulus occidentalis*] |
| Gene.3227 | 1.64 | Up | 0.007448 | Cytochrome P450 4c3 [*Metaseiulus occidentalis*] |
