# Supplementary material for: Comparative Transcriptome and Proteome Analysis of Heat Acclimation in Predatory Mite Neoseiulus barkeri
Source: Front Physiol. 2020 Apr 29;11:426. doi: 10.3389/fphys.2020.00426 (PMC7201100; doi:10.3389/fphys.2020.00426)
Supplement: TABLE S6 — Down-regulated proteins in HTAS of Neoseiulus barkeri proteomes. [file Table_6.DOCX]

Table S6 Down-regulated proteins in HTAS of *Neoseiulus barkeri* proteomes.

| **Protein ID** | **Mean Ratio HTAS/CS** | | **Up/Down-Regulation** | **P-value** | **NCBInr Description** |
| --- | --- | --- | --- | --- | --- |
| Gene.29407 | 0.48 | Down | | 0.01014 | - |
| Gene.8487 | 0.49 | Down | | 0.02239 | uncharacterized protein [*Metaseiulus occidentalis*] |
| Gene.44757 | 0.5 | Down | | 0.02505 | - |
| Gene.52 | 0.5 | Down | | 0.02247 | Maleylacetoacetate isomerase-like [*Metaseiulus occidentalis*] |
| Gene.40418 | 0.51 | Down | | 0.03109 | Cuticle protein 14-like [*Metaseiulus occidentalis*] |
| Gene.36028 | 0.52 | Down | | 0.02232 | PHD finger-like domain-containing protein 5A-like [*Maylandia zebra*] |
| Gene.18347 | 0.53 | Down | | 0.00526 | Calcineurin-like phosphoesterase domain-containing protein 1-like [*Metaseiulus occidentalis*] |
| Gene.49606 | 0.53 | Down | | 0.002967 | Ras-like GTP-binding protein Rho1-like [*Metaseiulus occidentalis*] |
| Gene.38129 | 0.56 | Down | | 0.004753 | Integrator complex subunit 11-like [*Metaseiulus occidentalis*] |
| Gene.16978 | 0.57 | Down | | 0.03866 | S phase cyclin A-associated protein [*Metaseiulus occidentalis*] |
| Gene.46114 | 0.57 | Down | | 0.01246 | uncharacterized protein [*Metaseiulus occidentalis*] |
| Gene.51467 | 0.57 | Down | | 0.01614 | Lysosomal aspartic protease [*Stegodyphus mimosarum*] |
| Gene.34109 | 0.58 | Down | | 0.01476 | Actin-dependent regulator of chromatin subfamily B member [*Metaseiulus occidentalis*] |
| Gene.4814 | 0.58 | Down | | 0.007803 | - |
| Gene.46417 | 0.58 | Down | | 0.001815 | Ribosomal L37ae protein family [*Acanthamoeba castellanii*] |
| Gene.42105 | 0.58 | Down | | 0.01266 | Nucleolar protein 14-like [*Metaseiulus occidentalis*] |
| Gene.18673 | 0.59 | Down | | 0.01226 | uncharacterized protein [*Metaseiulus occidentalis*] |
| Gene.18371 | 0.6 | Down | | 0.01604 | Vitellogenin-2-like [*Metaseiulus occidentalis*] |
| Gene.18894 | 0.63 | Down | | 0.001763 | Glutathione S-transferase 1 [*Metaseiulus occidentalis*] |
| Gene.6142 | 0.64 | Down | | 0.006399 | Ioleucine--tRNA ligase [*Metaseiulus occidentalis*] |
