# Supplementary material for: Comparative Transcriptome and Proteome Analysis of Heat Acclimation in Predatory Mite Neoseiulus barkeri
Source: Front Physiol. 2020 Apr 29;11:426. doi: 10.3389/fphys.2020.00426 (PMC7201100; doi:10.3389/fphys.2020.00426)
Supplement: TABLE S7 — Correlation between mRNAs and proteins with the same trend. [file Table_7.DOCX]

Table S7 Correlation between mRNAs and proteins with the same trend.

| **Correlation ID** | **Protein Sig.** | **DEPs** | **Gene Ratio (HTAS/CS)** | **Gene Sig.** | **DEGs** | **NCBInr description** |
| --- | --- | --- | --- | --- | --- | --- |
| Unigene4736_All | * | - | -5.19525 | 7.93E-09 | - | TRM112-like protein-like [*Metaseiulus occidentalis*] |
| Unigene4232_All | * | + | 1.043743 | 5.36E-06 | + | Mini-chromosome maintenance complex-binding protein-like *[Metaseiulus occidentalis]* |
| Unigene2364_All | * | - | -1.01601 | 0.002839 | - | Uncharacterized protein *[Metaseiulus occidentalis]* |
| CL237.Contig1_All | * | - | -1.80288 | 5.34E-23 | - | - |
| CL4033.Contig2_All | * | + | 1.249298 | 4.60E-05 | + | Guanine nucleotide-binding protein G(o) subunit alpha-like *[Metaseiulus occidentalis]* |
| Unigene2006_All | * | - | -1.84536 | 0.040738 | - | Conserved hypothetical protein *[Ixodes scapularis]* |
| CL1981.Contig2_All | * | - | -2.78115 | 1.67E-26 | - | Uncharacterized protein *[Metaseiulus occidentalis]* |
| CL2914.Contig1_All | * | + | 1.257249 | 2.97E-12 | + | Calcyclin-binding protein-like *[Metaseiulus occidentalis]* |
| Unigene5567_All | * | + | 1.838685 | 2.34E-12 | + | Uncharacterized protein *[Metaseiulus occidentalis]* |
| Unigene6011_All | * | - | -1.22589 | 0.000193 | - | Glucose-6-phosphate 1-epimerase-like *[Metaseiulus occidentalis]* |
| Unigene10676_All | * | - | -1.08483 | 1.94E-05 | - | Uncharacterized protein *[Metaseiulus occidentalis]* |
| CL1218.Contig3_All | * | - | -1.11194 | 0.01212 | - | Endoribonuclease Dcr-1-like *[Metaseiulus occidentalis]* |
| CL2077.Contig5_All | * | + | 3.736915 | 0.0001 | + | Cuticle protein 14-like *[Metaseiulus occidentalis]* |
| CL4197.Contig1_All | * | + | 1.034018 | 7.53E-06 | + | Uncharacterized protein *[Metaseiulus occidentalis]* |
| Unigene15778_All | * | + | 1.184841 | 4.16E-12 | + | Hsp90 co-chaperone *[Metaseiulus occidentalis]* |
| Unigene3945_All | * | - | -1.12777 | 0.001005 | - | Neuralized-like protein 4-like *[Metaseiulus occidentalis]* |
| CL2343.Contig2_All | * | + | 1.38791 | 5.42E-09 | + | - |
| Unigene14988_All | * | + | 0.999505 | 0.001033 | + | Uncharacterized protein *[Metaseiulus occidentalis]* |
| Unigene8302_All | * | - | -1.1223 | 0.002628 | - | - |
| CL472.Contig1_All | * | - | -1.24419 | 0.000814 | - | - |
| CL2189.Contig3_All | * | - | -1.14785 | 2.53E-05 | - | Uncharacterized protein *[Metaseiulus occidentalis]* |
| Unigene14121_All | * | - | -1.04496 | 0.000224 | - | CD9 antigen-like *[Metaseiulus occidentalis]* |
| Unigene3852_All | * | + | 1.051719 | 1.77E-07 | + | Cysteine and histidine-rich domain-containing protein 1-like *[Metaseiulus occidentalis]* |
| Unigene8517_All | * | + | 1.311004 | 9.02E-10 | + | DNA replication licensing factor mcm4-A-like *[Metaseiulus occidentalis]* |
| CL2893.Contig2_All | * | - | -1.12469 | 0.00052 | - | Leukotriene A-4 hydrolase-like *[Metaseiulus occidentalis]* |
| Unigene5353_All | * | - | -1.16182 | 1.16E-06 | - | Uncharacterized protein *[Metaseiulus occidentalis]* |
| Unigene7617_All | * | + | 1.402785 | 3.74E-23 | + | Uncharacterized protein *[Metaseiulus occidentalis]* |
| CL4004.Contig2_All | * | - | -1.12337 | 5.10E-05 | - | Endochitinase-like *[Metaseiulus occidentalis]* |
| CL15.Contig1_All | * | + | 1.302475 | 5.41E-11 | + | Kinesin-like protein KIF20A-like *[Metaseiulus occidentalis]* |
| Unigene365_All | * | + | 1.182221 | 5.91E-10 | + | - |
| CL2746.Contig1_All | * | + | 2.608638 | 1.09E-13 | + | Uncharacterized protein *[Metaseiulus occidentalis]* |
| Unigene12906_All | * | - | -4.13795 | 1.83E-05 | - | Ribosomal l37ae protein family *[Acanthamoeba castellanii]* |
| Unigene6038_All | * | - | -1.14875 | 7.35E-05 | - | Uncharacterized protein *[Metaseiulus occidentalis]* |
| CL738.Contig1_All | * | + | 1.338373 | 1.37E-14 | + | NEDD8 ultimate buster 1-like *[Metaseiulus occidentalis]* |
| CL4217.Contig2_All | * | - | -3.83866 | 7.10E-21 | - | - |
| CL3042.Contig1_All | * | - | -1.71399 | 5.23E-31 | - | Calpain-A-like *[Metaseiulus occidentalis]* |
| Unigene594_All | * | + | 3.743845 | 1.10E-65 | + | Uncharacterized protein *[Metaseiulus occidentalis]* |
| CL4252.Contig1_All | * | + | 4.040235 | 3.71E-05 | + | Isocitrate dehydrogenase [NADP], mitochondrial-like *[Metaseiulus occidentalis]* |
| CL4252.Contig2_All | * | - | -2.01691 | 7.15E-31 | - | - |
| CL1606.Contig1_All | * | - | -1.05834 | 0.004494 | - | - |
| Unigene8620_All | * | - | -1.78626 | 0.000324 | - | 4-coumarate-coa ligase 5-like *[Metaseiulus occidentalis]* |
| Unigene13780_All | * | + | 1.405195 | 3.35E-13 | + | Uncharacterized protein *[Metaseiulus occidentalis]* |
| CL2582.Contig2_All | * | - | -1.24451 | 1.58E-08 | - | Protein Bm6820, isoform A *[Brugia malayi]* |
| CL125.Contig1_All | * | + | 1.038148 | 0.01154 | + | Vitellogenin 1 *[Neoseiulus cucumeris]* |
| Unigene15598_All | * | + | 2.775761 | 0.000267 | + | Uncharacterized protein *[Metaseiulus occidentalis]* |
| CL2427.Contig2_All | * | - | -2.47627 | 1.02E-37 | - | Calcineurin-like phosphoesterase domain-containing protein 1-like *[Metaseiulus occidentalis]* |
| Unigene12306_All | * | + | 1.086746 | 7.04E-09 | + | ATP-dependent DNA helicase Q5-like *[Metaseiulus occidentalis]* |
| CL4314.Contig2_All | * | - | -1.35458 | 1.14E-05 | - | Atlastin, partial *[Stegodyphus mimosarum]* |
| Unigene11556_All | * | - | -3.19136 | 3.36E-24 | - | - |
| Unigene14119_All | * | + | 1.183167 | 5.44E-07 | + | Peptidyl-prolyl cis-trans isomerase FKBP4-like *[Metaseiulus occidentalis]* |
| Unigene8814_All | * | + | 1.030719 | 7.47E-05 | + | Uncharacterized protein *[Metaseiulus occidentalis]* |
| CL3569.Contig3_All | * | - | -1.26345 | 9.53E-07 | - | Golgi-associated plant pathogenesis-related protein 1-like *[Metaseiulus occidentalis]* |
| Unigene9132_All | * | + | 1.144864 | 1.58E-06 | + | TRAF-interacting protein-like *[Metaseiulus occidentalis]* |
| CL216.Contig5_All | * | - | -1.4905 | 2.05E-08 | - | Uncharacterized protein *[Metaseiulus occidentalis]* |
| CL4918.Contig2_All | * | - | -1.33033 | 6.26E-05 | - | - |
| CL1402.Contig1_All | * | - | -1.05865 | 0.001141 | - | Phospholipid scramblase 1-like *[Picoides pubescens]* |
| Unigene13212_All | * | + | 1.685491 | 1.59E-11 | + | - |
| CL1281.Contig4_All | * | - | -1.2203 | 1.47E-05 | - | Uncharacterized protein *[Metaseiulus occidentalis]* |
| Unigene12796_All | * | + | 1.027232 | 6.57E-11 | + | Sulfide:quinone oxidoreductase, mitochondrial-like *[Metaseiulus occidentalis]* |
| Unigene12060_All | * | - | -1.60062 | 7.74E-11 | - | Cysteine proteinase *[Polysphondylium pallidum]* |
| Unigene5630_All | * | + | 1.327809 | 0.000738 | + | Inositol oxygenase-like *[Metaseiulus occidentalis]* |
| CL2427.Contig3_All | * | + | 1.438164 | 0.013305 | + | - |
| CL854.Contig1_All | * | - | -1.23102 | 2.39E-06 | - | Choline dehydrogenase, mitochondrial-like *[Metaseiulus occidentalis]* |
| CL3262.Contig1_All | * | - | -1.5214 | 2.60E-08 | - | Cathepsin L-like *[Metaseiulus occidentalis]* |
| CL2893.Contig4_All | * | - | -1.70414 | 8.16E-08 | - | Leukotriene A-4 hydrolase-like *[Metaseiulus occidentalis]* |
| CL1418.Contig6_All | * | + | 2.386068 | 3.17E-33 | + | Heat shock 70 kda protein cognate 4-like *[Metaseiulus occidentalis]* |
| Unigene17_All | * | + | 4.133433 | 1.29E-65 | + | Putative integrase core domain protein *[Trichinella spiralis]* |
| CL1224.Contig2_All | * | - | -1.38144 | 5.32E-08 | - | - |
| CL1878.Contig4_All | * | - | -2.46215 | 0.00017 | - | - |
| Unigene2675_All | * | + | 1.0034 | 1.46E-05 | + | Importin subunit alpha-4-like *[Metaseiulus occidentalis]* |
| CL1385.Contig4_All | * | - | -1.76332 | 6.90E-17 | - | Troponin C-like *[Metaseiulus occidentalis]* |
| Unigene5662_All | * | - | -1.04451 | 2.59E-12 | - | GLIPR1-like protein 1-like *[Metaseiulus occidentalis]* |
| Unigene4260_All | * | + | 1.023416 | 9.47E-08 | + | Uncharacterized protein *[Metaseiulus occidentalis]* |
| CL889.Contig2_All | * | - | -2.26448 | 2.23E-18 | - | Uncharacterized protein *[Metaseiulus occidentalis]* |
| CL1299.Contig2_All | * | - | -2.83529 | 0.000454 | - | Cuticle protein 14-like *[Metaseiulus occidentalis]* |
| Unigene5631_All | * | + | 1.29938 | 9.45E-08 | + | Uncharacterized protein *[Metaseiulus occidentalis]* |
| CL2045.Contig4_All | * | - | -1.09104 | 9.05E-05 | - | - |
| CL295.Contig2_All | * | - | -1.01737 | 0.000287 | - | Uncharacterized protein *[Metaseiulus occidentalis]* |
| Unigene4207_All | * | - | -1.39008 | 0.000441 | - | - |
| CL2575.Contig3_All | * | + | 1.106715 | 0.011552 | + | Uncharacterized protein *[Metaseiulus occidentalis]* |
| Unigene14132_All | * | + | 1.001724 | 0.000111 | + | Protein LSM14 homolog B-B-like *[Metaseiulus occidentalis]* |
| CL1691.Contig3_All | * | - | -1.92396 | 5.96E-16 | - | Cytochrome P450 3A16-like *[Metaseiulus occidentalis]* |
| CL955.Contig2_All | * | - | -1.17897 | 0.001692 | - | Putative acid phosphatase 5-like *[Metaseiulus occidentalis]* |
| Unigene3883_All | * | - | -1.48064 | 4.08E-12 | - | PR-5-like protein *[Lysiphlebus testaceipes]* |
| Unigene1951_All | * | - | -1.43403 | 0.014125 | - | Putative chitinase 3, partial *[Stegodyphus mimosarum]* |
