# Supplementary material for: Comparative Transcriptome and Proteome Analysis of Heat Acclimation in Predatory Mite Neoseiulus barkeri
Source: Front Physiol. 2020 Apr 29;11:426. doi: 10.3389/fphys.2020.00426 (PMC7201100; doi:10.3389/fphys.2020.00426)
Supplement: TABLE S8 — Correlation between mRNAs and proteins with the opposite trend. [file Table_8.DOCX]

Table S8 Correlation between mRNAs and proteins with the opposite trend.

| **Correlation ID** | **Protein Sig.** | **DEPs** | **Gene Ratio (HTAS/CS)** | **Gene Sig.** | **DEGs** | **NCBInr description** |
| --- | --- | --- | --- | --- | --- | --- |
| CL1628.Contig2_All | * | - | 1.407119 | 0.010717 | + | Solute carrier family 22 member 5-like *[Metaseiulus occidentalis]* |
| Unigene2003_All | * | + | -1.69149 | 0.000198 | - | TNF receptor-associated factor 4-like *[Metaseiulus occidentalis]* |
| Unigene8357_All | * | + | -1.39929 | 1.83E-12 | - | Low-density lipoprotein receptor, putative *[Ixodes scapularis]* |
| Unigene14954_All | * | + | -1.6317 | 0.046479 | - | Peroxisomal carnitine O-octanoyltransferase-like *[Metaseiulus occidentalis]* |
| Unigene21141_All | * | - | 2.263924 | 0.02449 | + | Cathepsin L2 *[Capsaspora owczarzaki]* |
| CL1861.Contig1_All | * | + | -1.16203 | 5.21E-08 | - | Urocanate hydratase-like *[Metaseiulus occidentalis]* |
| CL3888.Contig1_All | * | + | -2.11228 | 0.001223 | - | Desumoylating isopeptidase 2-like *[Metaseiulus occidentalis]* |
| CL663.Contig2_All | * | + | -1.00858 | 9.91E-08 | - | - |
| CL3042.Contig4_All | * | + | -1.43406 | 4.20E-26 | - | Calpain-A-like *[Metaseiulus occidentalis]* |
| Unigene12388_All | * | - | 1.449723 | 3.21E-08 | + | Uncharacterized protein *[Metaseiulus occidentalis]* |
| CL4923.Contig1_All | * | - | 1.119838 | 4.61E-05 | + | Uncharacterized protein *[Metaseiulus occidentalis]* |
| CL4005.Contig3_All | * | - | 5.976255 | 5.13E-13 | + | Uncharacterized protein *[Metaseiulus occidentalis]* |
| CL3562.Contig1_All | * | + | -1.14497 | 0.000346 | - | Dual oxidase-like *[Metaseiulus occidentalis]* |
| CL783.Contig4_All | * | + | -5.91806 | 3.20E-13 | - | ATP-binding cassette sub-family B member 7 *[Metaseiulus occidentalis]* |
| Unigene10425_All | * | + | -1.13304 | 0.003275 | - | - |
| CL418.Contig3_All | * | + | -1.17996 | 5.25E-06 | - | Protein Skeletor, isoforms B/C-like *[Metaseiulus occidentalis]* |
| Unigene21689_All | * | - | 2.281317 | 0.040207 | + | Hypothetical protein *[Latrodectus hesperus]* |
| CL2142.Contig1_All | * | - | 1.029724 | 3.16E-07 | + | Glutamine synthetase-like isoform 2 *[Metaseiulus occidentalis]* |
| CL590.Contig8_All | * | + | -1.60838 | 5.09E-17 | - | FGGY carbohydrate kinase *[Metaseiulus occidentalis]* |
| CL2861.Contig1_All | * | - | 4.69253 | 1.48E-07 | + | Hypothetical protein *[Daphnia pulex]* |
| Unigene11698_All | * | - | 1.136002 | 7.95E-13 | + | Anaphase-promoting complex subunit 1 *[Metaseiulus occidentalis]* |
| CL4649.Contig1_All | * | + | -2.09333 | 0.040026 | - | Uncharacterized protein *[Metaseiulus occidentalis]* |
| CL593.Contig7_All | * | - | 1.119777 | 9.98E-05 | + | Rho gtpase-activating protein 190-like *[Metaseiulus occidentalis]* |
| Unigene6947_All | * | - | 1.219792 | 1.35E-06 | + | - |
| CL1590.Contig3_All | * | - | 1.298454 | 0.002648 | + | - |
| Unigene15329_All | * | + | -1.37477 | 1.28E-06 | - | Uncharacterized protein *[Metaseiulus occidentalis]* |
| CL4423.Contig2_All | * | + | -2.95866 | 0.004678 | - | Protein epsilon, partial *[Stegodyphus mimosarum]* |
| Unigene15574_All | * | + | -1.59167 | 1.29E-10 | - | Uncharacterized protein *[Metaseiulus occidentalis]* |
| CL3245.Contig2_All | * | + | -1.58442 | 1.94E-18 | - | Serine/threonine-protein kinase OSR1-like *[Metaseiulus occidentalis]* |
| CL4872.Contig2_All | * | - | 2.217149 | 0.041147 | + | Apolipoprotein D *[Chrysemys picta bellii]* |
| CL2420.Contig1_All | * | - | 1.453673 | 3.09E-07 | + | Inosine-uridine preferring nucleoside hydrolase, putative *[Ixodes scapularis]* |
| Unigene22681_All | * | + | -2.32497 | 0.036087 | - | Hypothetical protein *[Caenorhabditis brenneri]* |
| Unigene613_All | * | + | -1.58278 | 0.000864 | - | Uncharacterized protein *[Metaseiulus occidentalis]* |
| CL912.Contig2_All | * | + | -1.03022 | 0.001165 | - | - |
